# Whole-heart 4D flow can be acquired with preserved quality without respiratory gating facilitating clinical use

**DOI:** 10.1186/1532-429X-17-S1-Q17

**Published:** 2015-02-03

**Authors:** Mikael Kanski, Johannes Töger, Katarina Steding-Ehrenborg, Christos G Xanthis, Karin Markenroth Bloch, Einar Heiberg, Marcus Carlsson, Håkan Arheden

**Affiliations:** 1 Cardiac MR group Lund, Dept. of Clinical Physiology, Lund University, Lund, Sweden; 2 Dept. of Numerical Analysis, Center of Mathematical Sciences, Lund University, Lund, Sweden; 3 Dept. of Computer Science and Biomedical informatics, University of Thessaly, Lamia, Greece; 4 Philips Healthcare, Lund, Sweden; 5 Department of Biomedical Engineering, Faculty of Engineering, Lund University, Lund, Sweden

## Background

Respiratory gating is often used in 4D-flow acquisition to reduce motion artifacts. However, gating increases scan time. The aim of this study was to investigate if respiratory gating can be excluded from 4D flow acquisitions without affecting quantitative intracardiac parameters.

## Methods

Eight volunteers underwent CMR at 1.5T with a 5-channel coil (5ch). Imaging included 2D flows, and whole-heart 4D flow with and without respiratory gating (Resp(+), Resp(-)). Stroke volume (SV), particle-trace volumes, kinetic energy, and vortex-ring volume were obtained from 4D flow-data. These parameters were compared between 5ch Resp(+) and 5ch Resp(-).

## Results

**Figure 1:**
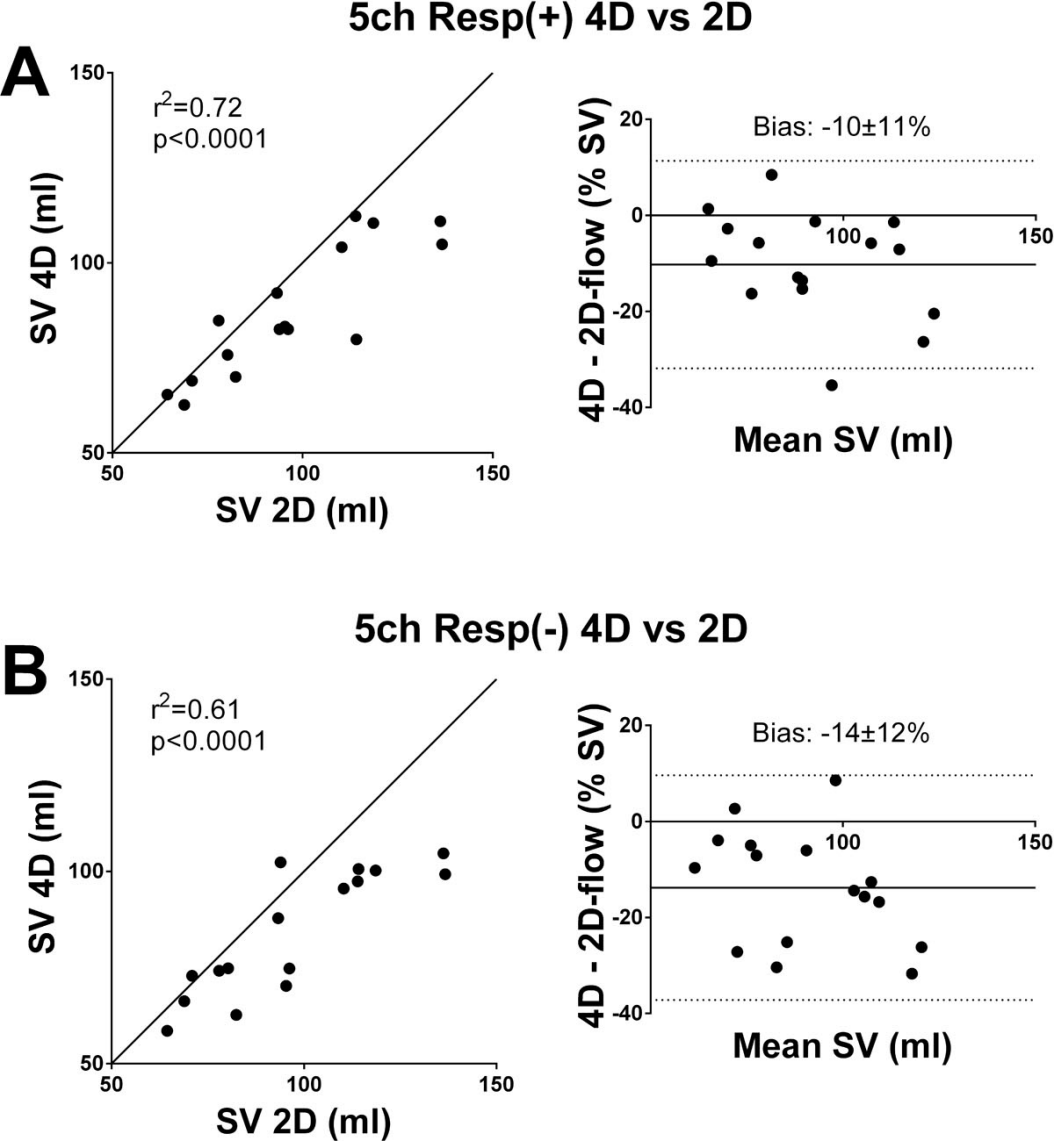
Comparison between SV from respiratory-gated 4D (Resp(+)) and non-gated 4D (Resp(-)) vs conventional 2D flow. Both SV deduced from aortic and main pulmonary arterial flow are included. The left column shows scatter plots of SV from 4D flow (ml) and 2D flow (ml). Solid lines show the line of identity. The right column shows the corresponding Bland Altman plots (bias±1.96SD). Panel A shows comparison between Resp(+) 4D and 2D. Panel B show the results for SV from 4D Resp(-). The bias and spread were similar for both methods.

Stroke volume from 4D flow was lower compared to 2D flow (5ch Resp(+) 86.9±17.0 vs 97.1±22.7, p=0.001; 5ch Resp(-) 83.9±16.0 vs 97.1±22.7, p<0.001), with no difference in bias (-10.3±11.0% vs -13.8±11.9%, p=0.16) (Figure 1). There was a good correlation between Resp(+) and Resp(-) for particle-trace derived volumes (R^2^=0.82, 1.6±12.4%), mean kinetic energy (R^2^=0.86, -4.3±12.4%), peak kinetic energy (R^2^=0.88, 0.7±16.9%), and vortex-ring volume (R^2^=0.70, -3.7±13.2%). Average scan duration for Resp(-) was shorter compared to Resp(+) (27±9 min vs 61±19 min, p<0.05).

## Conclusions

Whole-heart 4D flow can be acquired with preserved quality without respiratory gating, facilitating clinical use.

## Funding

This study was supported by the Swedish Research Council (2011-3916, 2012-4944); the Swedish Heart-Lung Foundation; Lund University Faculty of Medicine; Lund University Hospital, Lund, Sweden; and Region Skåne.

